# The carbonate concentration mechanism of *Pyropia yezoensis* (Rhodophyta): evidence from transcriptomics and biochemical data

**DOI:** 10.1186/s12870-020-02629-4

**Published:** 2020-09-15

**Authors:** Baoyu Zhang, Xiujun Xie, Xuehua Liu, Linwen He, Yuanyuan Sun, Guangce Wang

**Affiliations:** 1 Key Laboratory of Experimental Marine Biology, Center for Ocean Mega-Science, Institute of Oceanology, Chinese Academy of Sciences, Qingdao, China; 2 Laboratory for Marine Biology and Biotechnology, Qingdao National Laboratory for Marine Science and Technology, Qingdao, China

**Keywords:** Carbon concentrating mechanism, Enzyme activity, *Pyropia yezoensis*, Photosynthetic efficiency, Transcriptome

## Abstract

**Background:**

*Pyropia yezoensis* (Rhodophyta) is widely cultivated in East Asia and plays important economic, ecological and research roles. Although inorganic carbon utilization of *P. yezoensis* has been investigated from a physiological aspect, the carbon concentration mechanism (CCM) of *P. yezoensis* remains unclear. To explore the CCM of *P. yezoensis*, especially during its different life stages, we tracked changes in the transcriptome, photosynthetic efficiency and in key enzyme activities under different inorganic carbon concentrations.

**Results:**

Photosynthetic efficiency demonstrated that sporophytes were more sensitive to low carbon (LC) than gametophytes, with increased photosynthesis rate during both life stages under high carbon (HC) compared to normal carbon (NC) conditions. The amount of starch and number of plastoglobuli in cells corresponded with the growth reaction to different inorganic carbon (Ci) concentrations. We constructed 18 cDNA libraries from 18 samples (three biological replicates per Ci treatment at two life cycles stages) and sequenced these using the Illumina platform. De novo assembly generated 182,564 unigenes, including approximately 275 unigenes related to CCM. Most genes encoding internal carbonic anhydrase (*CA*) and bicarbonate transporters involved in the biophysical CCM pathway were induced under LC in comparison with NC, with transcript abundance of some *PyCA*s in gametophytes typically higher than that in sporophytes. We identified all key genes participating in the C4 pathway and showed that their RNA abundances changed with varying Ci conditions. High decarboxylating activity of PEPCKase and low PEPCase activity were observed in *P. yezoensis*. Activities of other key enzymes involved in the C4-like pathway were higher under HC than under the other two conditions. Pyruvate carboxylase (PYC) showed higher carboxylation activity than PEPC under these Ci conditions. Isocitrate lyase (ICL) showed high activity, but the activity of malate synthase (MS) was very low.

**Conclusion:**

We elucidated the CCM of *P. yezoensis* from transcriptome and enzyme activity levels. All results indicated at least two types of CCM in *P. yezoensis*, one involving CA and an anion exchanger (transporter), and a second, C4-like pathway belonging to the PEPCK subtype. PYC may play the main carboxylation role in this C4-like pathway, which functions in both the sporophyte and gametophyte life cycles.

## Background

Seaweeds are important marine photoautotrophs, playing an important part in global primary production and carbon sequestration [1–3].

The carbon in seawater exists in three forms: carbonic acid (H_2_CO_3_), bicarbonate ions (HCO_3_^−^) and carbonate ions (CO_3_^2−^). Approximately 90% of the total organic carbon in seawater is present as bicarbonate ion [4]. Due to the low amount of CO_2_ in seawater, most macroalgae have evolved a carbon concentrating mechanism (CCM) to utilize HCO_3_^−^ for maintaining high levels of growth [5–7]; these CCMs are increasingly important because rising CO_2_ in the air has led to rising levels of HCO_3_^−^ in water over the past few decades [8, 9]. Despite the ecological, economic and cultural importance of macroalgae, we know relatively little about their CCMs.

Various types of CCMs have been discovered in terrestrial plants and microalgae: biophysical, biochemical and basal CCM, respectively. Biophysical CCMs involve carbonic anhydrase (CA) and bicarbonate transporters (BCT). A biochemical CCM is also called the C4-like pathway. Three subtypes of C4 photosynthesis are recognized, based on the principal decarboxylating enzyme used in the bundle sheath: NADP-malic enzyme (NADP-ME), NAD-malic enzyme (NAD-ME) and phosphoenolpyruvate carboxykinase (PEPCK) [10]. Mitochondrial γ-CAs and NADH–ubiquinone oxidoreductase complex I are the main components in basal CCM [11, 12].

**Fig. 1:**
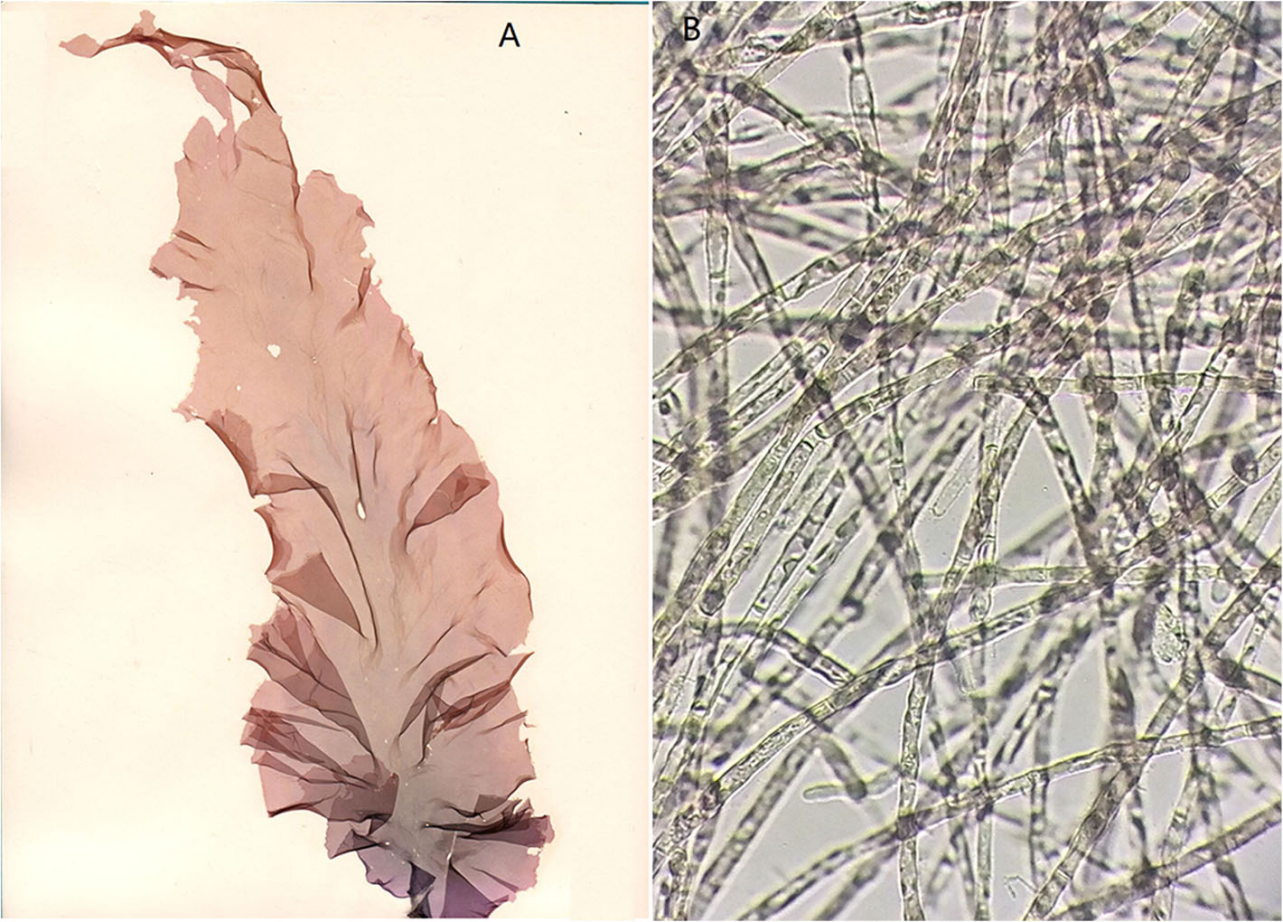
The photos of leafy thalli (gametophytes) and filamentous thalli (sporophytes). **a**. leafy thalli. **b**. filamentous thalli

Species of the genus *Pyropia* belong to the Rhodophyta are among the most economically important macroalgae in East Asia, including China, Japan and South Korea. The *Pyropia* life cycle involves a macroscopic leafy thallus phase (gametophyte) and a microscopic filamentous thallus phase (sporophyte) (Fig. 1) [13]. Due to its great economical, ecological and research value, *Pyropia* has been recognized as a model seaweed among marine plants [14, 15].

Extensive surveys have explored the physical reaction of *Pyropia* to rising CO_2_, indicating that elevated CO_2_ concentration (1000–1200 ppm) can enhance growth of *Pyropia yezoensis* [16, 17]; moreover, inorganic carbon (Ci) uptake styles differ between sporophytes and gametophytes. For instance, Yue et al. and Li et al. reported that gametophytes of *P. yezoensis* utilize HCO_3_^−^ via extracellular carbonate anhydrase (eCA), but exhibit weak ability to directly use HCO_3_^−^ [5, 18]. Luo et al. indicated that sporophytes absorb Ci through active transport of HCO_3_^−^ and CO_2_ [19]. By contrast, data from Expressed Sequence Tag (EST) and transcriptome studies suggest the possible existence of a C4-like pathway in *P. yezoensis* and *Pyropia haitanensis*, but whether this functions in *P. yezoensis* remains unknown [20–22].

To explore CCMs in *P. yezoensis*, we cultivated gametophytes and sporophytes under three different Ci conditions by adjusting the amount of NaHCO_3_ in artificial seawater. We tracked their growth, transcriptome and relative enzyme activities under different Ci conditions. Our results provide explanations for the divergence of CCMs between the two life stages of *P. yezoensis*.

## Results

### Physiological responses and change of pH under different Ci conditions

We tracked the pH of water and physiological characteristics of the two different *P. yezoensis* life cycle stages under culture conditions with different Ci.

**Fig. 2:**
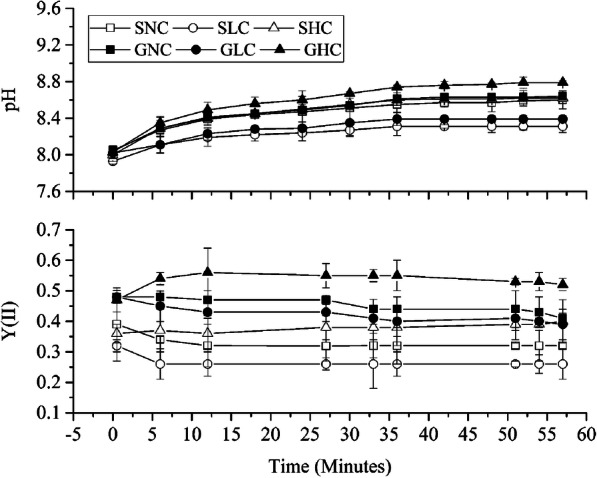
pH and YII changes in gametophytes and sporophytes of *P. yezoensis* under different Ci conditions. SNC, SHC and SLC indicate sporophytes cultivated under normal carbon (NC), high carbon (HC) and low carbon (LC) conditions, respectively. GNC, GHC and GLC indicate gametophytes cultivated under NC, HC and LC conditions, respectively. **a**. pH change in gametophytes and sporophytes under different Ci conditions. **b**. YII change in gametophytes and sporophytes under different Ci conditions. Results are represented as mean ± standard deviation

In sporophytes, pH ranged from 8.03 ± 0.03 to 8.64 ± 0.02 and 8.59 ± 0.03 under high carbon (HC) and normal carbon (NC) conditions, respectively. Under low carbon (LC), pH ranged from 8.00 ± 0.03 to 8.32 ± 0.03 (Fig. 2). Under LC, photosynthetic efficiency was around 17.75 ± 0.1% lower than that under NC, while it was enhanced by about 14.11 ± 0.08% under HC compared with NC, as indicated by changes in photosystem II (YII) (Fig. 2).

In gametophytes, the pH ranged from 8.04 ± 0.02 to 8.78 ± 0.03 under HC, from 8.02 ± 0.02 to 8.65 ± 0.04 under NC and from 8.02 ± 0.03 to 8.4 ± 0.02 under LC (Fig. 2). Photosynthetic efficiency was almost 5.91 ± 0.07% lower under LC than under NC, while it was enhanced by 16.77 ± 0.08% under HC compared with that under NC (Fig. 2).

### Transmission electron microscopy (TEM) observation of cells from the two life stages under three Ci conditions

**Fig. 3:**
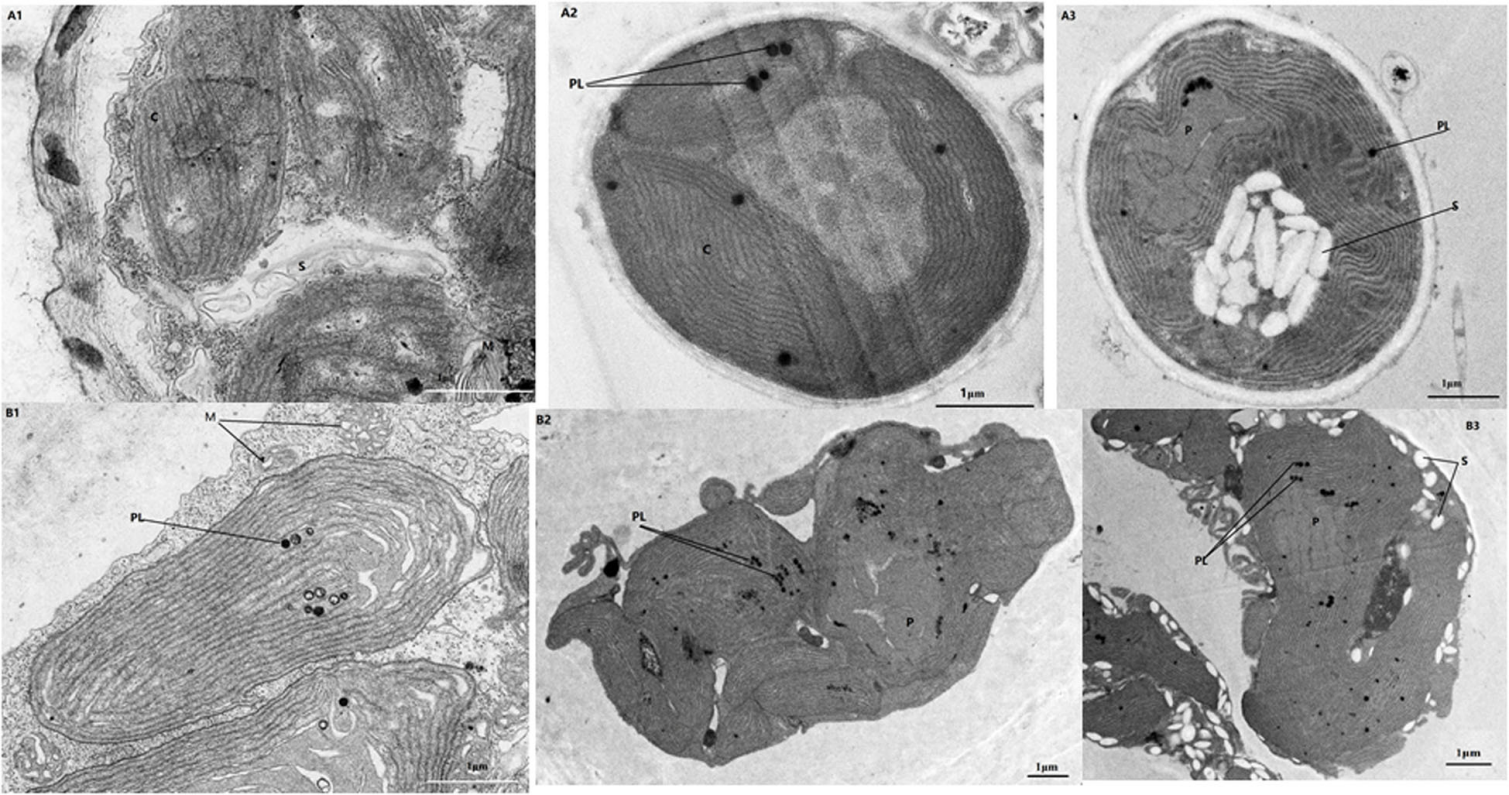
Transmission electron microscope images of sporophyte (**A**) and gametophyte (**B**) cells of *P. yezoensis* under three Ci conditions. Sporophytes cultivated under NC (A1), LC (A2) and HC (A3); gametophytes cultivated under NC (B1), LC (B2) and HC (B3). PL: plastoglobulus; C: chloroplast; M: mitochondrion; S: starch

Both gametophytes and sporophytes of *P. yezoensis* exhibited significant changes in the amount of starch and number of chloroplast-localized plastoglobuli, and even pyrenoid structure, with changing environmental conditions (Fig. 3). After culture for 54 h under LC, starch was undetectable in cells of both gametophytes and sporophytes, but the number of plastoglobuli in sporophytes increased from 1 to 2 in each cell under NC to 2–7 under LC. The number of plastoglobuli in gametophytes increased from 2 to 6 in each cell under NC to 2–20 under LC.

Under HC, cells of the two life stages had dramatically more starch than cells growing under NC, usually distributed between chloroplasts in sporophytes but outside of chloroplasts in gametophytes. Plastoglobuli obviously increased in these two life stages under HC, with their number increasing to 7–20 in each sporophyte cell and 10–40 in each gametophyte cell. Under NC, we observed a small amount of starch in the cells of sporophytes but barely any in gametophytes. Pyrenoid structure in gametophytes was loose and there were many ribosomes spread throughout the cytoplasm, while in sporophytes, pyrenoid structure was relatively tight and there were fewer ribosomes than in gametophytes.

### Illumina sequencing, de novo assembly and annotation

To explore the CCM of *P. yezoensis*, we cultivated gametophytes and sporophytes under different Ci conditions and constructed 18 cDNA libraries (generated from three biological replicates of the two life stages under the three treatments). Using Illumina Hiseq sequencing technology, each reaction can yield 2 × 150 bp independent reads from either end of a DNA fragment. We obtained high-quality clean reads accounting for more than 97.83% of the raw reads (Additional file 1, Table S1).

After assembling the high-quality clean reads from the high-throughput sequencing data, 182,564 unigenes were identified with a contig N50 size of 945 bp and average unigene size of 837 bp (Table 1). The length of these assembled unigenes ranged from 200 to 42,138 bp. Unigenes with lengths between 401 and 600 bp were predominant, comprising approximately 36.54% of the total number of unigenes; the next most abundant size class was 601–1000 bp, constituting 27% of the total unigenes (Additional file 2, Fig. S1). Both the number and the average length of unigenes were higher than those in reported transcriptome data. Yang [23] obtained 31,538 unigenes with an average length 419 nt, and Xie [22] obtained 24,575 unigenes with an average length of 645 bp in *P. haitanensis*. More recently, Wang obtained 34,465 unigenes in *P. haitanensis* [24].

**Table 1:** summary of Trinity assembly of the RNA-seq data of *P. yezoensis*

Total unigenes num.:	182,564
Total unigenes length:	152,921,448
Total isoform num.	202,495
Total isoform length	181,907,159
Average Unigene length:	837.631997546066
Largest unigene:	42,138
N50:	945

We performed BLAST analysis on all 182,564 unigenes using the following databases: NCBI non-redundant protein sequences (Nr), Swiss-Prot, Kyoto Encyclopedia of Genes and Genomes database (KEGG), and Clusters of Orthologous Groups of proteins (COG). We found 59,825 (32.77%), 59,327 (32.49%) and 53,110 (32.5%) unigenes in Swiss-Prot, Nr, and KEGG, respectively (Additional file 3, Table S2). These results might be due to a lack of complete genome information for *P. yezoensis.* Although genome sequences of *P. yezoensis* are freely available, they are either incomplete or lack annotation [25].

All these data indicated that the RNA-seq data met the quality standard for further analysis. After primary analysis, we identified around 275 unigenes related to CCM pathways.

### Induction of BCT and CA under different ci indicates the presence of a biophysical CCM

**Fig. 4:**
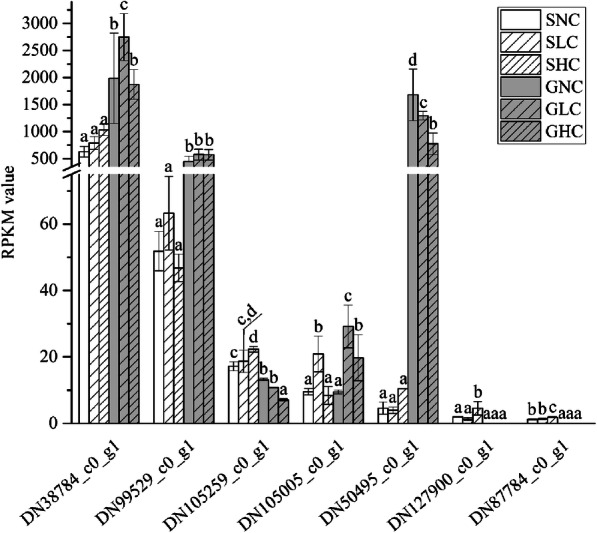
RPKM values of some CA unigenes in gametophytes and sporophytes of *P. yezoensis* under three Ci conditions. Data represent the mean ± standard deviation from three biological replicates. Means followed by same lowercase letters are not significantly different at *p* ≤ 0.05 by one-way ANOVA and Tukey’s test. Unigene IDs are DN38784_c0_g1, DN99529_c0_g1, DN105259_c0_g1, DN105005_c0_g1, DN50495_c0_g1, DN127900_c0_g1, and DN87784_c0_g1. SNC, SLC, SHC, GNC, GLC and GHC are as in Fig. 1

We identified a very large number of CA unigenes in this transcriptome. IDs for some of the *CA* unigenes, their identity in Nr and Swiss-Prot, and protein subtypes are listed in Additional file 4, Table S3. Three CA unigenes, DN38784_c0_g1 (beta-), DN 99529_c0_g1 (beta-) and DN105005_c0_g1 (alpha-), the former predicted to be located in the chloroplast and the other two in other cell positions (Additional file 5, Table S4), were upregulated under LC compared with NC. Transcripts of DN105005_c0_g1 increased by 2.08- and 1.1-fold in gametophytes and sporophytes under LC compared with NC, respectively. Such induction of specific CAs by low HCO_3_^−^ stress suggests an active biophysical CCM. Moreover, DN38784_c0_g1, DN 99529_c0_g1 and DN50495 _c0_g1 (alpha-), expression levels in gametophytes were significantly higher (over 10 times or even over 100 times) than those in sporophytes under the three Ci conditions, and the divergence was obvious (Fig. 4). In addition, some other CA unigenes, such as DN127900_c0_g1 (gamma-) and DN87784_c0_g1 (gamma-), predicted to be located in the mitochondrion (Additional file 6, Table S4), showed very low RPKM (reads per kilobase of exon model per million mapped reads) values in sporophytes and undetectable values in gametophytes under these three Ci conditions (Fig. 4).

Anion exchange proteins and ABC-transporters may play the role of bicarbonate transporters, as indicated from the transcriptome. Expression of DN101765_c1_g1, a putative Band 3 anion transporter sharing 58% amino acid sequence identity with its known counterparts from *Porphyridium purpureum* (KAA8497371) and *Gracilariopsis chorda* (PXF50105), was increased by 0.4- and 0.5-fold in sporophytes and gametophytes, respectively, under LC compared with NC. Expression of a putative anion exchange protein, DN107803_c0_g1, was increased 0.36- and 0.65-fold in sporophytes and gametophytes, respectively, under LC compared with NC.

In addition, transcripts of several ABC-transporters (105272_c0_g1, 109304_c3_g1) were upregulated under LC compared with NC in the two life cycles; moreover, the RPKM values of these unigenes in sporophytes were significantly higher than those in gametophytes.

### Increased transcript abundance and enzyme activity of C4-like enzymes under different Ci indicates an active biochemical CCM

Key genes involved in the C4-pathway were all identified in this transcriptome, and some unigenes encoding these key enzymes are listed in Additional file 4, Table S3. The subcellular location of key enzymes involved in the C4-pathway was predicted using different prediction servers and the results are shown in Additional file 5, Table S4.

BlastX search showed that DN107354_c0_g1 had 100% amino acid sequence identity with *PEPC* in *P. yezoensis* (accession no. AIT70077); it was predicted to be located in neither the mitochondrion nor the chloroplast, and may be located in the cytosol (Additional file 5, Table S4). We identified DN101889_c0_g3 and DN101912_c0_g1 as transcripts of *PEPCK* due to very high amino acid identity (64 to 77%), and predicted them to be targeted to mitochondria. DN141134_c0_g1, DN14534_c0_g1 and DN87488_c0_g1 were identified as putative unigenes of pyruvate phosphate dikinase (*PPDK*); the former two were predicted to be located in the chloroplast, and the latter was predicted to be located in the mitochondrion (Table S4). DN74954_c0_g1, DN34191_c0_g1 and DN50799_c0_g1 encode malate dehydrogenase (*MDH*) and were predicted to target to the mitochondria, another cellular position and the chloroplast, respectively (Table S4). We also identified a few unigenes encoding *ME*, such as DN75319_c0_g1 and DN53078_c0_g1. DN15039_c0_g1 and DN105351_c0_g1 are pyruvate carboxylase (*PYC*) unigenes, and the former was predicted to be located in the mitochondria while the latter localized to another position in the cell (Table S4).

**Fig. 5:**
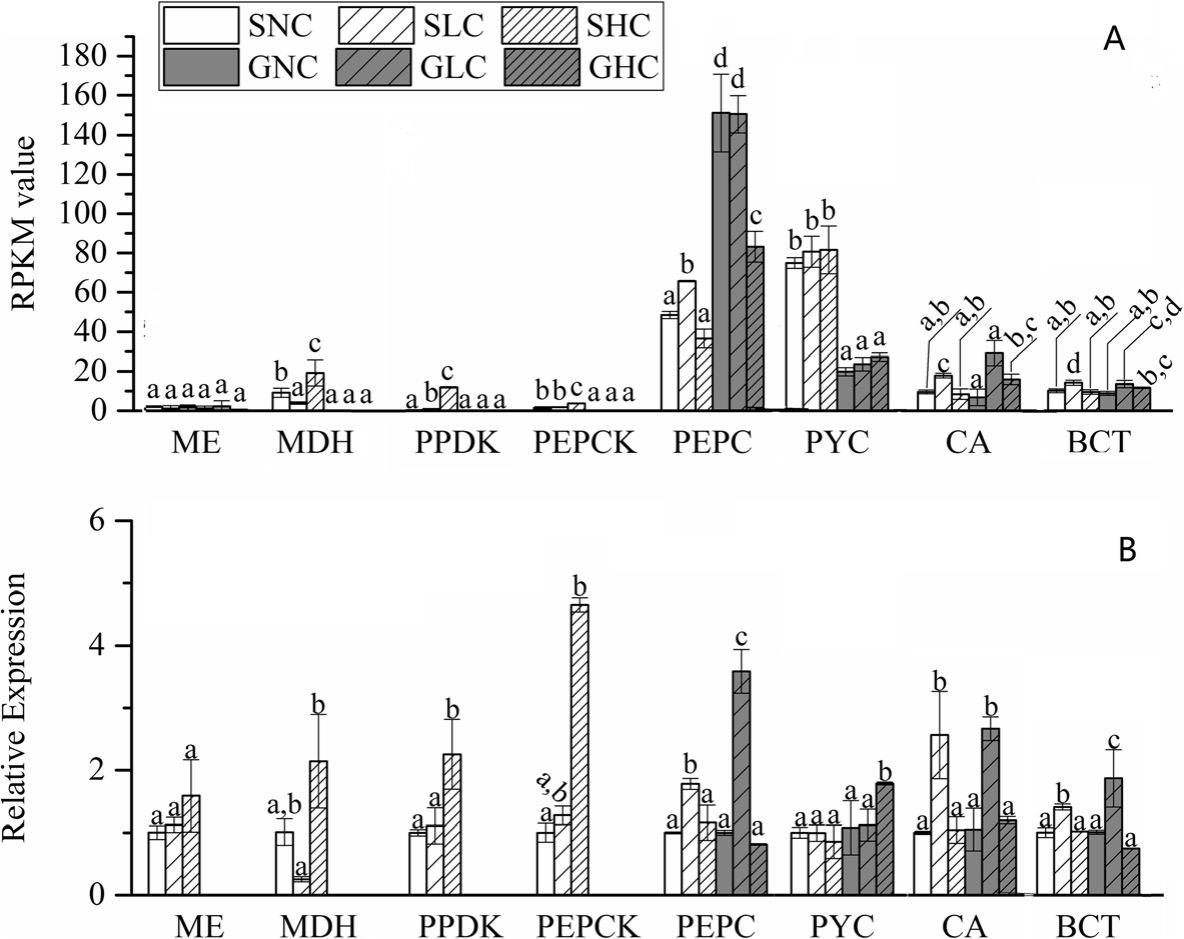
Expression levels of some key genes involved in biochemical CCM based on RNA-Seq assays (**A**) and relative expression levels verified by qPCR (**B**) in gametophytes and sporophytes of *P. yezoensis* under three Ci conditions. Data represent the mean ± standard deviation from three biological replicates. Means followed by same lowercase letters are not significantly different at *p* ≤ 0.05 by one-way ANOVA and Tukey’s test. ME (DN53078_c0_g1), MDH (DN74954_c0_g1)9, PPDK (DN14534 _c0_g1), PEPCK (DN101889_c0_g3), PEPC (DN107354_c0_g1), PYC (DN105351_c0_g1), CA (DN105005_c0_g1), BCT (DN101765_c1_g1)

The expression of these unigenes involved in biochemical CCM under different Ci conditions is indicated in RPKM and shown in Fig. 5a. Under HC conditions, the expression of *MDH*, *PPDK* and *PEPCK* unigenes was upregulated, in sporophytes, moreover, the divergency from expression is obvious, although the RPKM value was low (Fig. 5a). The *MDH* (DN74954_c0_g1), *PPDK* (DN14534_c0_g1) and *PEPCK* (DN101889_c0_g3) unigene were induced by 1.1-, 32- and 1.45-fold, respectively, under HC compared with NC in sporophytes. The expression of these three genes in gametophytes was so low that they were almost undetectable. Meanwhile, the expression of *PEPC* was downregulated under HC compared with NC in these two life stages, and decreased 0.25- and 0.45-fold in sporophytes and gametophytes (Fig. 5a). Under LC conditions, the expression of *PEPC* and *PPDK* was upregulated, and increased 0.35- and 1.56-fold, respectively, compared with that under NC conditions in sporophytes. While the expression of *MDH* was downregulated and decreased 0.57-fold in sporophytes. The expression of *ME* and *PYC* unigenes showed no obvious difference under different Ci concentrations in the two life stages (Fig. 5a).

Changes in gene expression for individual targets were validated using real-time fluorescent quantitative reverse - transcription PCR (qRT-PCR) (Fig. 5b). Among eight detected unigenes, most genes showed a similar expression tendency to that revealed by transcriptome data, except for the *PEPC* unigene (DN107354c0g1). This was upregulated in gametophytes under LC compared with NC, while the transcriptome indicated no divergence between LC and NC conditions.

**Fig. 6:**
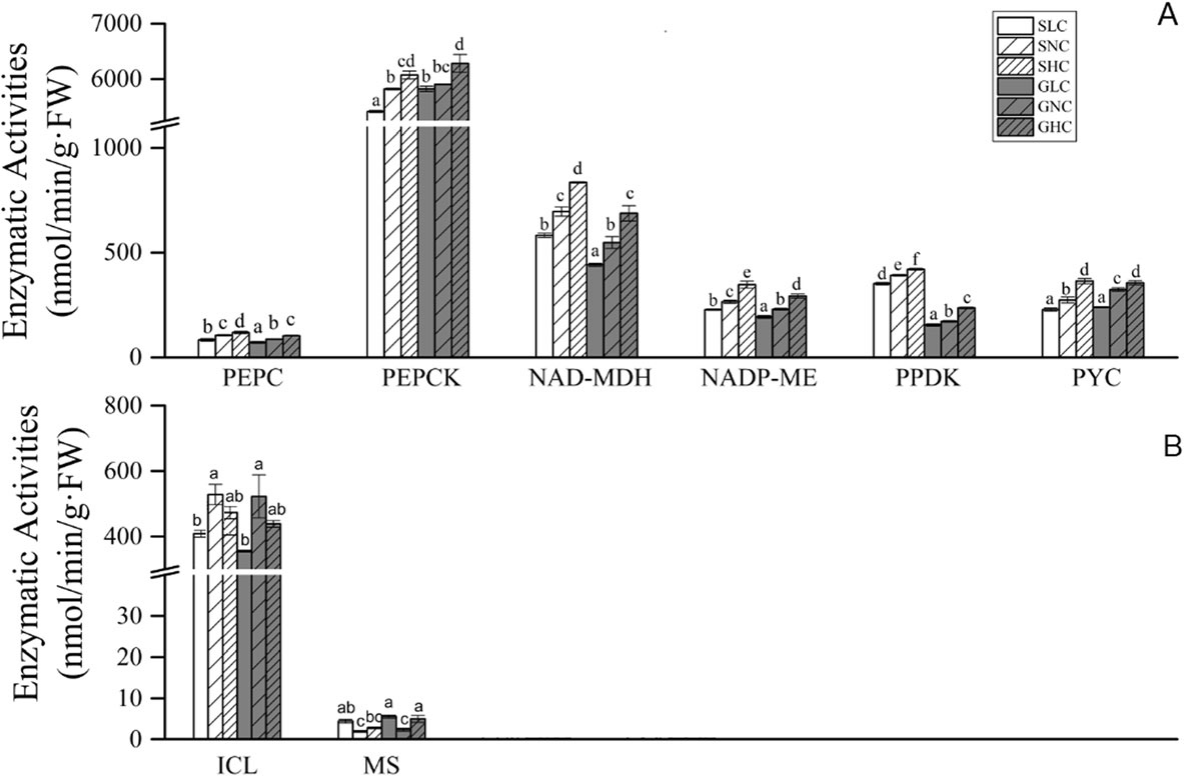
Activities of key enzymes involved in biochemical CCM (**A**) and the glyoxylate cycles (**B**) in gametophytes and sporophytes of *P. yezoensis* under different Ci conditions. Data represent the mean ± standard deviation from three biological replicates. Means followed by same lowercase letters are not significantly different at *p* ≤ 0.05 by one-way ANOVA and Tukey’s test. SNC, SLC, SHC, GNC, GLC and GHC are as in Fig. 1

We detected the activity of key enzymes involved in this pathway under the different Ci conditions (Fig. 6a). PEPC enzyme activity under HC was 119.3 and 103.9 nmol/min/g FW in sporophytes and gametophytes, respectively, higher than under LC and NC conditions. PEPCK showed very high decarboxylating activity in the two life cycles, greater than 5400 nmol/min/g FW. The enzyme activity of PEPCK under HC in gametophytes was a little higher than that in sporophytes, 6076.2 and 6238.7 nmol/min/g FW, respectively. Enzyme activity of PPDK under HC was higher than that under LC and NC. Moreover, PPDKase activity in sporophytes was about onefold higher than that in gametophytes. Reduction activity of NAD-MDH, which reduces oxaloacetate (OAA) into malate (MAL), was also detected; it was commonly higher in sporophytes than in gametophytes and was usually higher under HC relative to LC and NC: 835.6 and 688.5 nmol/min/g FW in sporophytes and gametophytes under HC, respectively. NADP-MEase activity in sporophytes was higher than that in gametophytes under HC, at 347.7 and 293.3 nmol/min/g FW, respectively. PYCase activity in gametophytes was a little higher than that in sporophytes under NC, at 323.7 and 274.5 nmol/min/g FW, respectively, while it was similar under HC in both life stages, at 356.8 and 364.6 nmol/min/g FW.

We also detected the activity of some key enzymes involved in the glyoxylate cycle (Fig. 6b). ICL and MS, the key enzymes involved in the glyoxylate cycle, showed dramatically different activities. The highest activity of ICL was observed under NC, at about 520 nmol/min/g FW in the two life stages. The lowest activity of ICL was 355 and 423 nmol/min/g FW in the gametophytes and sporophytes, under LC. The activity of MS was very low and around 3 nmol/min/g FW under three Ci conditions.

## Discussion

### Physical characteristics of the two life cycles under different Ci conditions

The effective photochemical quantum yield of photosystem II (YII) is directly related to the CO_2_ assimilation rate and represents the photosystem II state [26, 27]. As shown in Fig. 1, YII in gametophytes was higher than that in sporophytes under the same culture conditions. Huan (2018) studied the response of the photosynthetic reaction in the two life stages of *P. yezoensis* to increasing CO_2_, and found that YII in gametophytes was enhanced as CO_2_ increased; however, there were no significant differences in YII of sporophytes [28]. Wang (2019) reported that net photosynthetic rates (Pn) of thalli gametophytes were about 2.9 times those of conchocelis sporophytes of *P. haitanensis* at pH 8.0 [29]. All these results suggest that gametophytes of *Pyropia* usually have a higher photosynthetic rate than sporophytes under the same culture conditions. Interestingly, LC had more effect on sporophytes than on gametophytes, indicated by reduced photosynthetic efficiency (YII) under LC conditions.

Among the conditions affecting photosynthesis in aquatic plants is the hydrogen ion concentration of the growth environment. The pH of the medium determines the ratios of H_2_CO_3_, HCO_3_^−^ and CO_3_^−^, and then influences photosynthetic rate [30]. Since LC had a more obvious negative effect on YII in sporophytes than in gametophytes, pH change under LC culture was comparatively less for sporophytes relative to gametophytes. Under HC conditions, the photosynthetic efficiency of gametophytes was higher than that of sporophytes, so the pH of HC medium rose more quickly for gametophytes, accordingly. These results indicated that the reactions of sporophytes and gametophytes to the change of Ci concentration are different; sporophytes are more sensitive to the LC relative to gametophytes, while properly elevated Ci (such as 4 mM HCO_3_^−^ in seawater) enhances the photosynthetic efficiency of photosystem II in gametophytes more than in sporophytes.

The physiological state of algae is closely related to its metabolites. When *P. yezoensis* was cultured under LC, starch was consumed, as shown in Fig. 3. He et al. (2002) reported that starch in macroalgae was consumed first when macroalgae were in a hungry state [31]. When *P. yezoensis* was cultivated under HC conditions, more starch was observed (Fig. 3). Plastoglobuli, also called osmiophilic globuli, are plastid-localized lipoprotein particles [32–34]. Interestingly, we observed that the number of plastoglobuli changed in the two different life stages under HC and LC compared NC. The reason for this phenomenon corresponds to the role of plastoglobuli. It has been proved that plastoglobuli play an active role in metabolic and stress-response pathways, and are a metabolic intersection between different plastid compartments [35, 36].

### Biophysical CCM in the two different life cycles of *P. yezoensis*

Facing the phenomenon of rising global CO_2_, researchers are paying more attention to the inorganic carbonate utilization of macroalgae, including *Pyropia*. Work has been conducted to explore the physiological reaction to rising CO_2_, the pH compensation point, and the physiological reaction after adding different types of inhibitors [19, 37, 38]. The pH compensation point of gametophytes and sporophytes of *P. haitanensis* is pH 9.9 and 9.95, respectively, and of gametophytes of *P. yezoensis* is pH 9.65 [5, 19]. High pH compensation point corresponds to the presence of CCMs [39]. However, despite the above inferences, we still lack direct molecular evidence. In this study, we found a large number of unigenes encoding CA and some unigenes encoding anion transporter protein, which maybe play roles in transporting HCO_3_^−^ into cells from the external environment. Since HCO_3_^−^ is not freely permeable across the lipid bilayer of biological membranes, it is either transported by membrane transporter proteins or obtained in the form of CO_2_, which is then converted by periplasmic CA [40].

HCO_3_^−^ uptake in the two different *Pyropia* life stages has been elucidated by physiological reactions to different types of inhibitors, such as acetazolamide (AZ), eCA inhibitor, ethoxyzolamide (EZ), iCA inhibitor and an inhibitor of the HCO_3_^−^ transporter, 4,4′-diisothiocyanato-stilbene-2,2′-disulfonic acid (DIDS). Luo and his coworkers (2002) examined the inhibitory effect of AZ, DIDS and vanadate, an inhibitor of ATPase associated with the plasma membrane, on sporophytes of *P. haitanensis*. They showed inhibitory rates of 25.3 and 71.3%, respectively, after adding AZ and vanadate, and inferred that eCA is not an important part of Ci uptake in *P. haitanensis* conchocelis, with most Ci absorption occurring through active transport of HCO_3_^−^ and CO_2_ [19]. Later, Li and his coworkers (2016) suggested that the gametophytes of *P. yezoensis* show active HCO_3_^−^ uptake by studying their reaction to enhanced CO_2_ in the atmosphere [5]. Recently, Wang (2019) studied the physiological reaction to Ci utilization between gametophytes and sporophytes of *P. haitanensis* and found that the Pn of thallus was significantly inhibited by AZ and EZ. For conchocelis, inhibition by EZ was greater than that by AZ. Inhibition of conchocelis by DIDS was greater than that of thallus. All these results indicate that iCA and eCA play essential roles in HCO_3_^−^ utilization in gametophytes, while iCA is more important than eCA in sporophytes. At the same time, the absorption of HCO_3_^−^ via the DIDS-sensitive anion transport protein is less important in gametophytes than in sporophytes [29].

Limitations of subcellular location software and servers prevented us from defining the eCA unigenes; thus, we could not determine their RNA expression levels under different Ci conditions. We detected a higher abundance of some chloroplast-targeted CA genes, for instance DN38784_c0_g1 and DN50495_c0_g1, in gametophytes than in sporophytes (Fig. 4), which suggested that biophysical CCMs play a more important role in gametophytes relative to sporophytes.

We identified some unigenes encoding anion exchange proteins and ABC-transporters, and these were upregulated under LC compared with NC. Moreover, the abundance of these genes encoding bicarbonate transporters was higher in sporophytes relative to gametophytes. These data are in agreement with evidence from inhibitory experiments that anion exchange proteins and ABC- transporters make a great contribution to HCO_3_^−^ transportation in sporophytes of *P. yezoensis*.

BCTs are divergent within the microalgae and macroalgae. In *Macrocystis pyrifera* (*Phaeophyta*), anion exchange protein plays the main role in bicarbonate uptake, while in microalgae, such as *Nannochloropsis oceanica* and *Chlamydomonas reinhardtii,* ABC-transporters play an essential role in HCO_3_^−^ transportation [41–43]. In *Phaeodactylum tricornutum*, the solute carrier family is the HCO_3_^−^ transporter [44].

### Biochemical CCM in *P. yezoensis*

Besides the C3 pathway, a C4-like pathway might exist in *Pyropia*. Fan et al. (2007) constructed an EST library of *P. haitanensis* sporophytes and found abundant PEPCK ESTs; they primarily inferred that a C4-like pathway might exist in sporophytes of *P. haitanensis* [21]. Later, genes involved in the C4-like pathway were identified in *P. yezoensis*, except *PPDK* [20]. Xie and his coworkers (2013) obtained the transcriptomes of gametophytes and sporophytes of *P. haitanensis* and found some key genes involved in the C4-pathway, but no unigenes encoding *PPDK* or *MDH*. They inferred that the C4-like pathway plays a role in sporophytes, according to RNA relative expression levels of *PEPCK* and *PEPC* [23]. In fact, there has not been enough direct evidence so far to support this opinion.

In our work, we not only found all the key genes involved in the C4-like pathway, including unigenes encoding *PPDK*, but we also detected the activity of these enzymes under different Ci conditions. At the transcript level, we found that each gene involved in the C4-like pathway had a few unigenes (Table S3), which may indicate that most genes belong to multicopy gene families. As Fig. 5 shows, unigenes involved in the C4-like pathway showed different responses when Ci conditions shifted from NC to HC/LC. Although the RNA abundance of some unigenes corresponding to *PPDK* or *PEPCK* genes was very low in the two life stages, there was obvious divergence in expression when Ci conditions shifted from NC to HC/LC.

At the enzyme activity level, key enzymes involved in the C4-like pathway showed similar tendencies, with higher activity under HC than under the other Ci conditions. From these results, we primarily suggest that the expression of these key genes under different Ci conditions does not correspond to relative enzyme activity.

High PEPCKase decarboxylation activity but low PEPCase activity was observed in this work. This situation has also been observed in other macroalgae. Xu (1991) reported this phenomenon in brown *Laminaria japonica* [45]. Later, Shao (2019) also found very high PEPCK decarboxylation activity under HC conditions (0.1 M KHCO_3_) in *Saccharina japonica*, but they did not detect PEPCase activity [46]. He (2013) reported that PEPCKase showed higher decarboxylation activity but lower carboxylation activity in *P. haitanensis* [47]. Kremer and Küppers found that PEPCase activities were very low or undetectable in some red, green and brown macroalgae [48].

Since low PEPCase activity exists in most macroalgae, we investigated another carboxylating enzyme – PYC. PYC functions in non-photosynthetic organisms, but in recent years, *PYC* genes have been found in various aquatic phototrophs, such as *C. reinhardtii* and *Chlorella variabilis* [49, 50]. Tsuji and his coworkers identified a plastid-located PYC and proposed that EhPYC1 contributes to active beta-carboxylation in *Emiliania huxleyi*, indicating a novel pathway for the production of C4 compounds [50]. In our work, PYCase showed higher carboxylating activity than PEPCase, thus providing the OAA for PEPCKase decarboxylation. That is to say, PYC plays the major carboxylation role in biochemical CCM in *P. yezoensis*. This is the first report of this to our knowledge.

OAA and MAL are also parts of the peroxisome glyoxylate cycle, at which carbohydrates are synthesized from C2 compounds [51, 52]. The key enzyme activities of the glyoxylate cycle, ICL and MS, were investigated. Higher ICL activity and lower MS activity indicated that the contribution of the glyoxylate cycle generating MAL is weak. However, NAD-MDHase showed high reduction activity in this study, and generated MAL. MAL in the cytosol can enter into the mitochondrion for use in other pathways, such as the TCA cycle and C4-like CCM pathway.

**Fig. 7:**
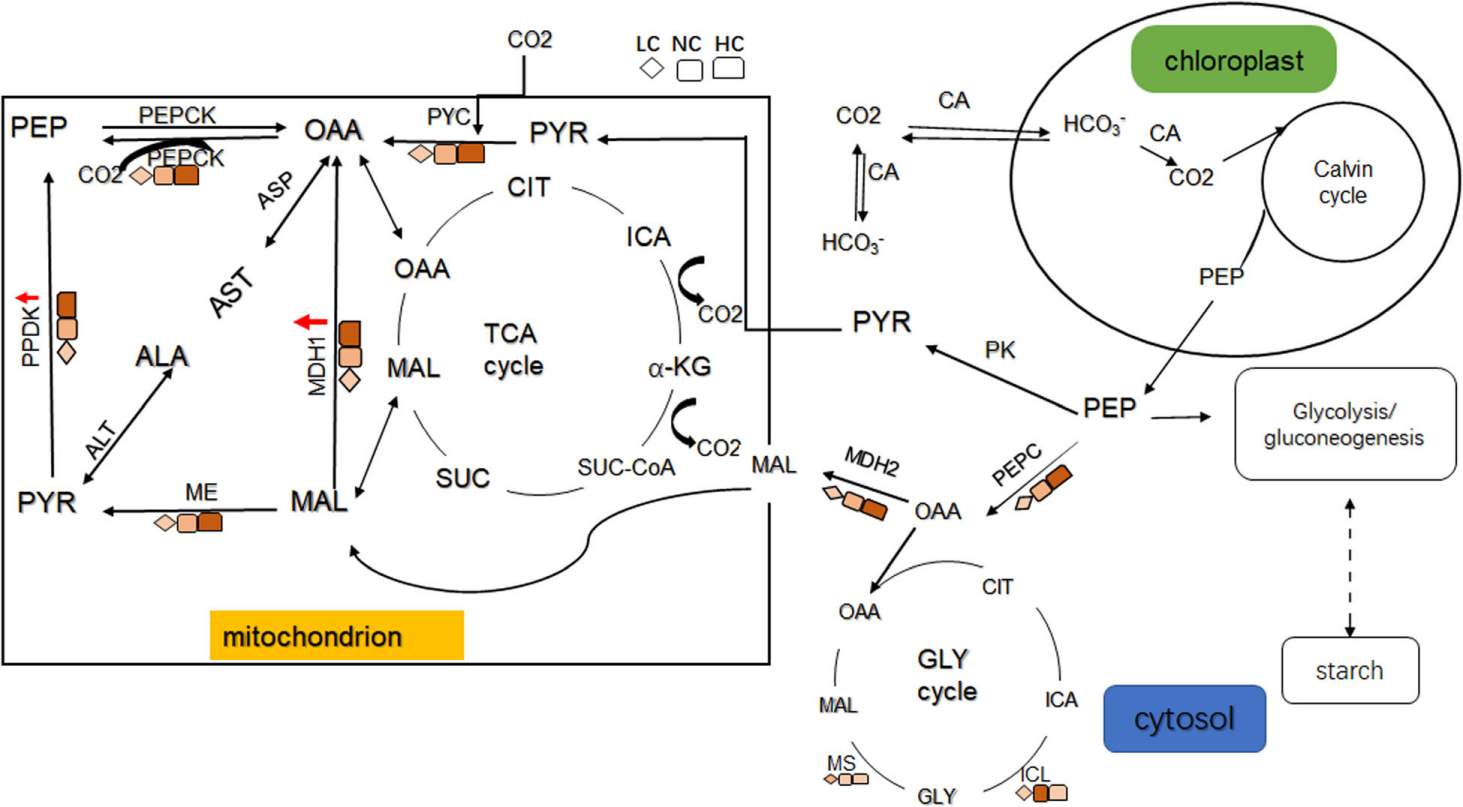
Diagram of the putative biochemical CCM pathway in *P. yezoensis* based on predicted subcellular location, enzymic activity and the pathway indicated by the transcriptome. Solid lines represent direct action, and dotted lines represent indirect action. Red arrow indicate that the expression of the gene was upregulated under HC conditions. Enzyme activity is indicated in different colors, and the deeper the color, the higher the enzyme activity. CIT:citrate; ICA: isocitrate; SUC: succinate; GLY: glyoxylate; α- KG:α-ketoglutarate

Combining our predicted subcellular location results, enzymic activity and the putative C4 pathway generated from the transcriptome, we constructed an overview of the biochemical CCM of *P. yezoensis* (Fig. 7). Of course, the precise subcellular location needs to be proved by location experiments. We primarily suggest that the C4-like pathway might be located in the mitochondrion and belong to the PEPCK subtype. Some enzymes involved in this pathway, such as PEPC and MDH, have cytosol-located isoforms and may play roles in the cytosol. The C4-like pathway not only plays a role in sporophytes, but also plays a role in gametophytes under NC and HC conditions, unlike the previous hypothesis.

## Conclusions

The CCM in *P. yezoensis* includes biophysical CCM and biochemical CCM, and they play an essential role for the growth of *P. yezoensis*. The two CCMs perform different roles according to environmental Ci concentrations. The biophysical CCM plays a more important role in gametophytes than in sporophytes, and the C4-like pathway also plays a role under high Ci conditions, explaining why gametophytes have higher photosynthesis rate than sporophytes. Moreover, PYC plays the major carboxylation role in biochemical CCM in *P. yezoensis*.

## Methods

### Algal materials

Wild resources of *Pyropia yezoensis* (Ueda) M. S. Hwang & H. G. Choi was originally collected in Qingdao, Shandong Province, China, and they are permitted by local government to collect. Then sporophytes of *P. yezoensis* were stored in the algae collection in the Institute of Oceanology, Chinese Academy of Sciences, Qingdao, China, and gametophytes of this species were generated from sporophytes under laboratory conditions. Before incubating in artificial seawater with different dissolved Ci concentrations, thallus gametophytes and filamentous sporophytes were cultured in Provasoli’s enriched seawater medium at 18 °C under 12 h light:12 h dark at 30 μmol/m^2^/s.

Artificial seawater (g/L: NaCl 20.758, NaSO_4_ 3.477, CaCl_2_ 1, KCl 0.587, MgCl_2_·6H_2_O 9.359, NaHCO_3_ 0.17, KBr 00845, H_3_BO_3_ 0.0225, NaF 0.0027, SrCl·6H_2_O 0.0214) was used for NC conditions. There was no NaHCO_3_ in artificial seawater representing LC and 4 mM HCO_3_^−^ (0.34 g/L) in artificial seawater with HC. NaHCO_3_ was added to the artificial seawater after autoclaving, and the pH was then adjusted to around 8.1.

### Transcriptome sampling, library construction, sequencing and analysis

Gametophytes and sporophytes were cultured in the three media mentioned above (LC, NC, HC) in a Multi-cultivator MC 1000-OD (Photon Systems Instruments, Drasov, Czech Republic) with cool white light (30 μmol photons/m^2^/s) at 18 °C. Fresh materials (0.05 g) were inoculated into 80 mL medium with three biological replicates for each Ci concentration. To avoid disturbance from outside carbon sources, media were bubbled continuously with mixed gas of high purity nitrogen and oxygen (4:1). After culturing for 54 h, 18 samples were collected for transcriptomic analyses. Total RNA was extracted from each sample using Trizol reagent (Invitrogen, Thermo Scientific, USA). The concentration and purity of total RNA were assessed with spectrophotometers (NanoDrop-1000, Thermo Scientific, USA). Strand-nonspecific transcriptome libraries were prepared using a TruseqTM RNA sample prep Kit (Illumina, USA) and sequenced for 2 × 150 bp runs (paired-end) using an Illumina HiSeq 2000 at Biozeron Biotech. Co. Ltd., China.

After sequencing, the raw reads datasets were evaluated using Trimmomatic software (http://www.usadellab.org/cms/uploads/supplementary/Trimmomatic). Adapter sequences and lower-quality reads (shorter than 30 bp or quality score lower than 20) were filtered to obtain high-quality mRNA sequencing reads (Additional file 2, Table S1). The high-quality clean reads from all samples were assembled using Trinity software V2.2.0 (http://trinityrnaseq.sourceforge.net/) to produce an assembly gene set. All of the raw reads generated in this study have been deposited in China national GeneBank (https://db.cngb.org/cnsa/) with accession code CNP0000880.

The assembled gene set was used for BLASTX search (BLAST+ 2.7.1, E Value ≤1e-5) against the NR, Swiss-prot and KEGG databases for functional annotation. Gene expression under each experimental condition was measured as the number of aligned reads to annotated genes determined by Cufflinks (version 2.0.4) and normalized to RPKM values.

### Measurements of photosynthetic parameters and pH

pH, chlorophyll fluorescence of PSII (YII), maximum quantum yield (Fv/Fm), and dissolved CO_2_ and O_2_ concentrations were detected in real time in a flat-panel photobioreactor (Photobioreactor FMT 150 from Photon System Instruments). Culturing conditions were the same as those of the Multi-cultivator MC 1000-OD bioreactor, with 0.4 g fresh weight (FW) of *P. yezoensis* gametophytes or sporophytes added to 800 mL medium. Three biological replicates were conducted.

### qRT-PCR assay

The relative expression levels of genes listed in Table S5 from above mentioned cultures were measured by qRT-PCR. The protocol for qRT-PCR was performed by standard methods (Roche, Switzerland) as described previously [53], with Eukaryotic initiation factor 4A (EIF4A, TRINITY_DN83248_c0_g1) as an internal reference gene. Triplicate qRT-PCRs were performed for each sample. The relative expression of each gene under HC/LC conditions was determined by comparing with NC condition in each group, the sporophyte group and the gametophyte group. Primer pairs used for qRT-PCR analyses are listed in Additional file 6, Table S5.

### Statistical analysis

All the results in this study are shown as mean values ± standard deviation (*n* = 3). The data were firstly analyzed using one-way ANOVA and then Tukey’s test was used for post hoc analysis at the α = 0.05 significance level. All analyses were carried out using SPSS 18.0 (SPSS Inc., Chicago, Il, USA).

### The prediction of subcellular localization of proteins

To better understand the biochemical CCM pathway of *P. yezoensis*, information of subcellular location of putative proteins involved in this pathway is necessary. Four programs, SignalP, ChloroP, Mitoprot, and TargetP, were used to predict subcellular localization. The SignalP 5.0 server (http://www.cbs.dtu.dk/services/SignalP/) predicts the presence of signal peptides and the location of their cleavage sites in proteins from Archaea, Bacteria and Eukarya. ChloroP (http://www.cbs.dtu.dk/services/ChloroP/) predicts the presence of chloroplast transit peptides (cTP) in protein sequences and the location of potential cTP cleavage sites. MitoProt calculates the N-terminal protein region that can support a mitochondrial targeting sequence and the cleavage site [54]. The TargetP-2.0 server (http://www.cbs.dtu.dk/services/TargetP/) predicts the presence of N-terminal presequences: signal peptide, mitochondrial transit peptide, chloroplast transit peptide or thylakoid luminal transit peptide. Results from the four programs were pooled, and locations with higher scores or the same prediction results were chosen as the predicted localization for a particular protein. Unigenes encoding the key enzymes involved in the biochemical CCM pathway are listed in Additional file 5, Table S4.

### Measurement of the enzymatic activity involved in C4-like pathway and glyoxylate cycle

Enzymatic activities of PEPC, PEPCK, PPDK, NADP-ME, NAD-MDH, PYC, MS and ICL were measured using quantification kits (Keming Biotech, China) according to the user’s manual. In the protocol, about 100 mg fresh algal materials were ground on ice, and 1 mL extraction buffer from the respective kit was added. The mixture was stirred to homogeneity and centrifuged at 4 °C, 10,000 *g* for 10 min. Supernatants were incubated on ice for further enzyme assays. Enzyme activities were determined using a UV-1800 spectrophotometer by measuring the change of absorbance at 340 nm over 5 min in total volumes of 0.2 mL and in triplicate. The enzyme activity unit was defined as nmol NAD(P)H oxidation or NAD(P) + reduced per minute per gram fresh weight. The MS activity was determined by measuring the change of absorbance at 412 nm according to the protocol of kit.

### Transmission Electron microscopy

Samples were collected in a centrifuge tube after being cultivated for 54 h. Samples were then fixed in a solution of 4% glutaraldehyde with 0.01 M phosphate buffer, pH 7.4, at 4 °C and then centrifuged for 10 min, 5000 g at room temperature. Pellets were washed with phosphate buffer, and then post-fixed with 1% osmium tetroxide (Ted Pella INC, California, USA) in phosphate buffer, pH 7.4, for 1.5 h before washing three times with 0.01 M phosphate buffer at 4 °C. The samples were dehydrated in a series of ethanol from 30 to 100%, infiltrated with acetone (Tieta, Laiyang, China) and epoxy resin (SPI − CHEM, USA) mixture, and embedded and polymerized in epoxy resin. Sections were cut with a Leica EM UC7 ultramicrotome (Leica Microsystems, Germany), stained with citrate and examined with a transmission electron microscope (HT7700, Hitachi, Tokyo, Japan).

## Supplementary information

**Additional file 1: Table S1.** Statistics of quality control on RNA-seq data of gametophytes and sporophytes samples of *P. yezoensis* under different Ci conditions.

**Additional file 2: Figure S1.** Sequence length distribution of transcriptome.

**Additional file 3: Table S2.** Summary of *P. yezoensis* transcriptome.

**Additional file 4: Table S3.** The identity value and subtype of some unigenes of *P. yezoensis* involved in biochemical and biophysical CCM.

**Additional file 5: Table S4.** Predicted subcellular localization of some unigenes which encoding the key enzymes involved in biochemical and biophysical CCM in *P.yezoensis*. The analysis is based on predictions from various computational programs. Abbreviations: Y: Yes; **-,** not detected; C, chloroplast; M, mitochondrion; O, other; SP, signal peptide.

**Additional file 6: Table S5.** Primers used for qRT-PCR.

## Data Availability

All of the datasets supporting the results of this article are included within the article and its additional files. The raw reads of transcriptome in this study can be accessed with the link below: http://db.cngb.org/cnsa/project/CNP0000880/reviewlink/.

## Abbreviations

ABC transporter: ATP-binding cassette transporter
AZ: Acetazolamide
BCT: Bicarbonate transporters
CA: Carbonic anhydrase
eCA: Extracellular carbonate anhydrase
EST: Expressed sequence tag
CCM: Carbon concentration mechanism
iCA: Internal CA
Ci: Inorganic carbon
HC: High carbon
ICL: Isocitrate lyase
LC: Low carbon
MDH: Malate dehydrogenase
ME: Malic enzyme
MS: Malate synthase
NC: Normal carbon
PEPC: Phosphoenolpyruvate carboxylase
PEPCK: Phosphoenolpyruvate carboxykinase
PPDK: Pyruvate phosphate dikinase
PYC: Pyruvate carboxylase
RPKM: Reads Per kilobase of exon model per million mapped reads
TEM: Transmission electron microscopy
