# Supplementary figures and images for: The carbonate concentration mechanism of Pyropia yezoensis (Rhodophyta): evidence from transcriptomics and biochemical data
Source: BMC Plant Biol. 2020 Sep 15;20:424. doi: 10.1186/s12870-020-02629-4 (PMC7491142; doi:10.1186/s12870-020-02629-4)

Fig. 1


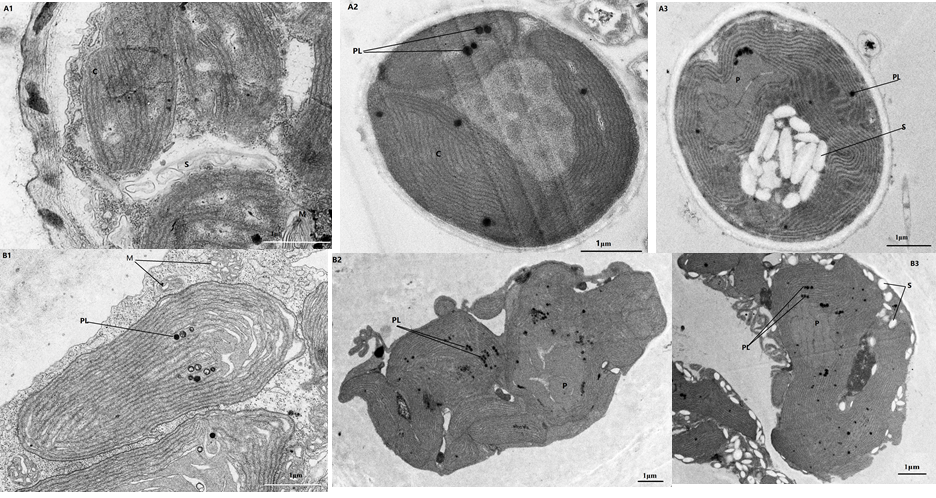


Fig. 2





Fig. 3

Fig. 4
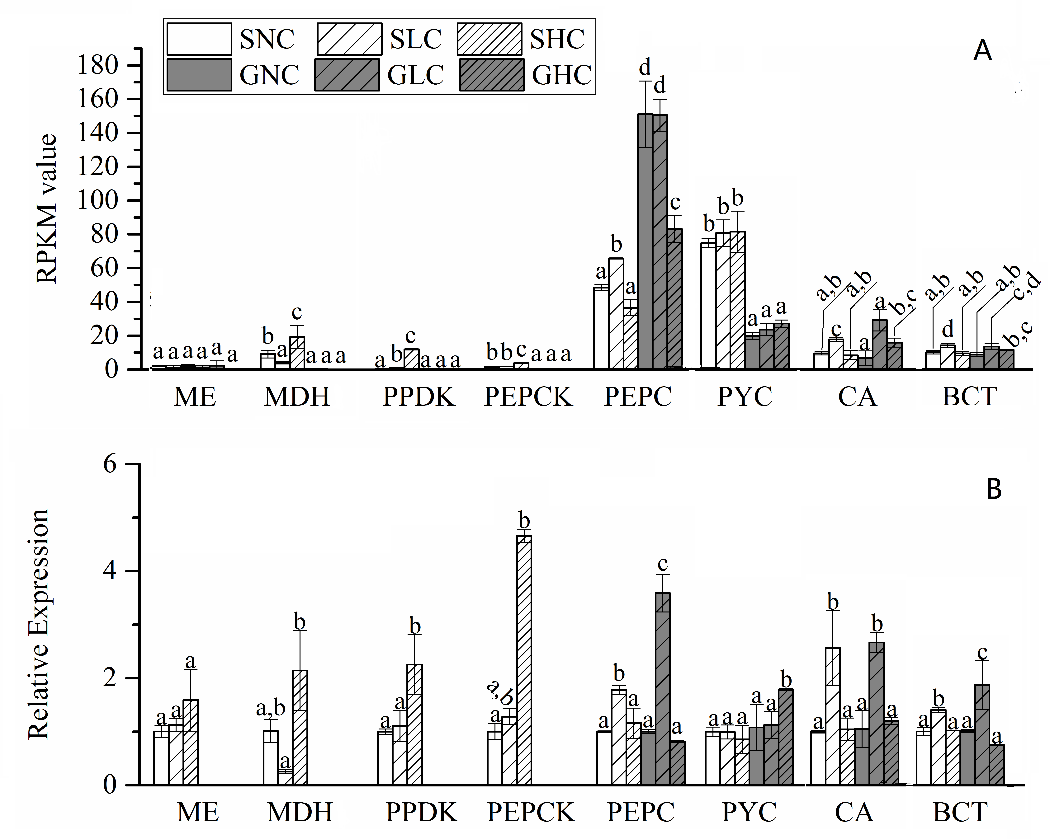


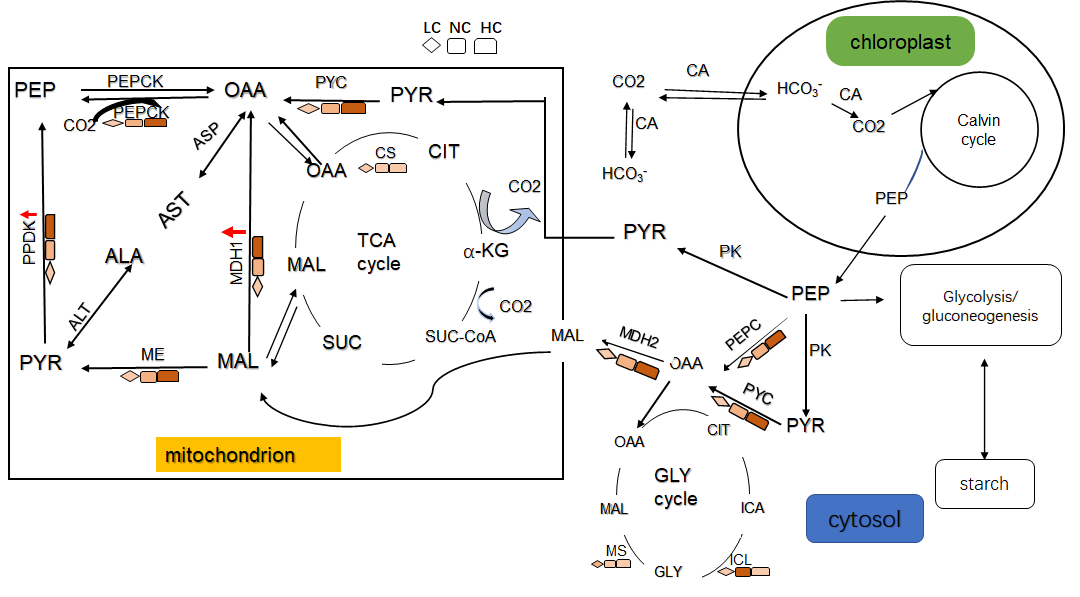


Fig. 6

Supplement: Supplementary file 1 — Additional file 1: Table S1. Statistics of quality control on RNA-seq data of gametophytes and sporophytes samples of P. yezoensis under different Ci conditions. [file 12870_2020_2629_MOESM1_ESM.docx]

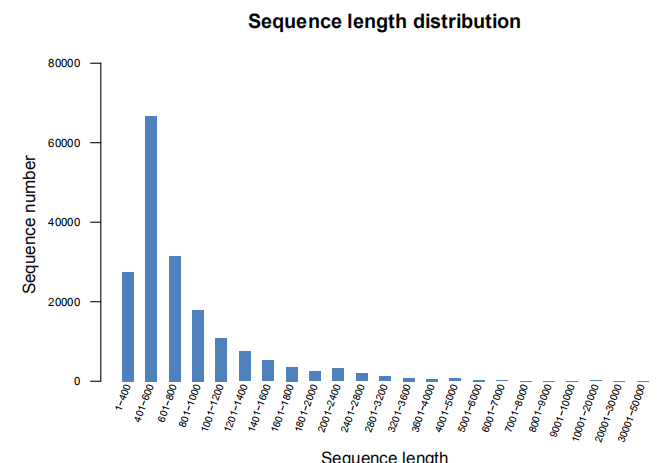

Supplement: Supplementary file 2 — Additional file 2: Figure S1. Sequence length distribution of transcriptome. [file 12870_2020_2629_MOESM2_ESM.docx]
