# Supplementary material for: The carbonate concentration mechanism of Pyropia yezoensis (Rhodophyta): evidence from transcriptomics and biochemical data
Source: BMC Plant Biol. 2020 Sep 15;20:424. doi: 10.1186/s12870-020-02629-4 (PMC7491142; doi:10.1186/s12870-020-02629-4)
Supplement: Supplementary file 3 — Additional file 3: Table S2. Summary of P. yezoensis transcriptome. [file 12870_2020_2629_MOESM3_ESM.docx]

| Item | number |
| --- | --- |
| Unigenes with annotation | 182564 |
| Annotated in Nr | 59327 |
| Annotated in GO | 11199 |
| Annotated in COG | 30245 |
| Annotated in KEGG | 53110 |
| Annotated in Swiss-prot | 59825 |
