# Supplementary material for: The carbonate concentration mechanism of Pyropia yezoensis (Rhodophyta): evidence from transcriptomics and biochemical data
Source: BMC Plant Biol. 2020 Sep 15;20:424. doi: 10.1186/s12870-020-02629-4 (PMC7491142; doi:10.1186/s12870-020-02629-4)
Supplement: Supplementary file 4 — Additional file 4: Table S3. The identity value and subtype of some unigenes of P. yezoensis involved in biochemical and biophysical CCM. [file 12870_2020_2629_MOESM4_ESM.docx]

| TableS3. The identity value and subtype of some unigenes involved in biochemical and biophysical CCM | | | | | subtype |
| --- | --- | --- | --- | --- | --- |
| Enzyme codes | putative protein | Unigene No. | Swissprot_to phsp_%-Similarity | NR_to phsp_%-Similarity |  |
| EC2.7.9.1 | Pyruvate orthophosphate dikinase(PPDK) | TRINITY_DN141134_c0_g1 | 74% | 76% |  |
|  |  | TRINITY_DN14534_c0_g1 | 70% | 61% |  |
|  |  | TRINITY_DN87488_c0_g1 | 72% | 69% |  |
| EC4.1.1.31 | Phosphoenolpyruvate carboxylase (PEPC) | TRINITY_DN107354_c0_g1 | 60% | 59% |  |
|  |  | TRINITY_DN134009_c0_g1 | 68% | 74% |  |
|  |  | TRINITY_DN130243_c0_g1 | 98% | 54% |  |
| EC4.1.1.49 | Phosphoenopyruvate carboxykinase (PEPCK) | TRINITY_DN101912_c0_g1 | 77% | 77% |  |
|  |  | TRINITY_DN101889_c0_g3 | 75% | 64% |  |
| EC1.1.1.40 | Malic enzyme (ME) | TRINITY_DN75319_c0_g1 | 63% | 62% |  |
|  |  | TRINITY_DN107480_c0_g1 | 65% | 64% |  |
|  |  | TRINITY_DN175824_c0_g1 | 68% | 66% | NADP- |
|  |  | TRINITY_DN53078_c0_g1 | 60% | 55% | NAD- |
| EC1.1.1.37(EC 1.1.1.82) | Malate dehydeogenase (MDH) | TRINITY_DN74954_c0_g1 | 78% | 77% |  |
|  |  | TRINITY_DN106212_c0_g1 | 73% | 72% |  |
|  |  | TRINITY_DN50799_c0_g1 | 55% | 50% | NAD- |
|  |  | TRINITY_DN34191_c0_g1 | 73% | 73% | NAD- |
| EC2.1.6.2 | Alanine aminotransferase (ALT) | TRINITY_DN107143_c0_g1 | 63% | 65% |  |
|  |  | TRINITY_DN127408_c0_g1 | 68% | 69% |  |
|  |  | TRINITY_DN100482_c0_g1 | 71% | 68% |  |
| EC2.1.6.1 | Aspartate aminotranferase (AST) | TRINITY_DN100628_c0_g1 | 100% | 99% |  |
|  |  | TRINITY_DN102918_c0_g1 | 98% | 85% |  |
|  |  | TRINITY_DN102005_c0_g1 | 70% | 69% |  |
| EC6.4.1.1 | Pyruvate carboxylase (PYC) | TRINITY_DN105351_c0_g1 | 69% | 67% |  |
|  |  | TRINITY_DN15039_c0_g1 | 70% | 69% |  |
|  | Bicarbonate transporter（BCT） | TRINITY_DN101765_c1_g1 | 49% | 58% |  |
|  | anion exchange family | TRINITY_DN107803_c0_g1 | 48% | 57% |  |
| EC4.2.1.1 | carbonic anhydrase (CA) | TRINITY_DN38784_c0_g1 | 71% | 69% | beta |
|  |  | TRINITY_DN99529_c0_g1 | 53% | 61% | beta |
|  |  | TRINITY_DN105259_c0_g1 | 45% | 43% | beta |
|  |  | TRINITY_DN105005_c0_g1 | 53% | 37% | beta |
|  |  | TRINITY_DN50495_c0_g1 | 50% | 52% | alpha |
|  |  | TRINITY_DN126328_c0_g1 | 47% | 53% | alpha |
|  |  | TRINITY_DN84778_c0_g1 | 42% | 44% | alpha |
|  |  | TRINITY_DN127900_c0_g1 | 47% | 59% | gama |
|  |  | TRINITY_DN87784_c0_g1 | 56% | 65% | gama |
