# Supplementary material for: The carbonate concentration mechanism of Pyropia yezoensis (Rhodophyta): evidence from transcriptomics and biochemical data
Source: BMC Plant Biol. 2020 Sep 15;20:424. doi: 10.1186/s12870-020-02629-4 (PMC7491142; doi:10.1186/s12870-020-02629-4)
Supplement: Supplementary file 5 — Additional file 5: Table S4. Predicted subcellular localization of some unigenes which encoding the key enzymes involved in biochemical and biophysical CCM in P.yezoensis. The analysis is based on predictions from various computational programs. Abbreviations: Y: Yes; -, not detected; C, chloroplast; M, mitochondrion; O, other; SP, signal peptide. [file 12870_2020_2629_MOESM5_ESM.docx]

| **Table S4. Subcellular localization of *P.yezoensis* related to biophysical CCM, the C4-like pathway. The analysis was based on predictions from various computational programs (Methods). Abbreviations: Y, Yes; -, not detected; C, chloroplast; M, mitochondrion; O, other; SP, signal peptide.** | | | | | | | | | | |
| --- | --- | --- | --- | --- | --- | --- | --- | --- | --- | --- |
| **Gene name** | **Unigene ID** | **SignalP** | **ChloroP** | | | **Mitoprot** | | **TargetP** | | **Sum Prediction** |
|  |  | **SP** | **Score** | **CTP** | **Score** | **MTP** | **Probability** | **result** | **Score** |  |
| **Biophysical CCM** | |  |  |  |  |  |  |  |  |  |
| **BCT** | **TRINITY_DN101765_c1_g1** | **-** | **0.0099** | **-** | **0.463** | **-** | **0.098** | **other** | **0.790** | **O** |
| **anion exchange family** | **TRINITY_DN107803_c0_g1** | **-** | **0.0005** | **-** | **0.454** | **Y** | **0.723** | **other** | **0.999** | **M** |
| **CA** | **TRINITY_DN38784_c0_g1** | **-** | **0.0016** | **Y** | **0.523** | **-** | **0.031** | **C** | **0.991** | **C** |
|  | **TRINITY_DN99529_c0_g1** | **-** | **0.0016** | **-** | **0.465** | **-** | **0.071** | **other** | **0.842** | **O** |
|  | **TRINITY_DN126328_c0_g1** | **Y** | **0.643** | **-** | **0.487** | **-** | **0.199** | **SP** | **0.822** | **C** |
|  | **TRINITY_DN84778_c0_g1** | **-** | **0.0018** | **-** | **0.463** | **-** | **0.015** | **other** | **0.991** | **O** |
|  | **TRINITY_DN105259_c0_g1** | **-** | **0.0076** | **Y** | **0.516** | **Y** | **0.909** | **other** | **0.727** | **M** |
|  | **TRINITY_DN50495_c0_g1** | **-** | **0.0054** | **Y** | **0.559** | **-** | **0.452** | **C** | **0.765** | **C** |
|  | **TRINITY_DN105005_c0_g1** | **-** | **0.007** | **Y** | **0.539** | **Y** | **0.775** | **other** | **0.961** | **O** |
|  | **TRINITY_DN87784_c0_g1** | **-** | **0.0028** | **-** | **0.452** | **Y** | **0.727** | **M** | **0.682** | **M** |
|  | **TRINITY_DN127900_c0_g1** | **Y** | **0.0006** | **-** | **0.446** | **Y** | **0.829** | **M** | **0.691** | **M** |
| **Biochemical CCM (C4-like Pathway)** | |  |  |  |  |  |  |  |  |  |
| **MDH** | **TRINITY_DN74954_c0_g1** | **-** | **0.0098** | **-** | **0.469** | **Y** | **0.991** | **M** | **0.831** | **M** |
|  | **TRINITY_DN106212_c0_g1** | **-** | **0.0007** | **Y** | **0.503** | **Y** | **0.968** | **M** | **0.838** | **M** |
|  | **TRINITY_DN50799_c0_g1** | **-** | **0.0158** | **Y** | **0.546** | **-** | **0.020** | **C** | **0.536** | **C** |
|  | **TRINITY_DN34191_c0_g1** | **-** | **0.0005** | **-** | **0.459** | **-** | **0.111** | **other** | **1.000** | **O** |
| **ME** | **TRINITY_DN75319_c0_g1** | **-** | **0.162** | **Y** | **0.513** | **-** | **0.021** | **other** | **0.638** | **O** |
|  | **TRINITY_DN107480_c0_g1** | **-** | **0.0004** | **-** | **0.456** | **Y** | **0.999** | **M** | **0.618** | **M** |
|  | **TRINITY_DN175824_c0_g1** | **-** | **0.0025** | **-** | **0.437** | **-** | **0.252** | **other** | **0.999** | **O** |
|  | **TRINITY_DN53078_c0_g1** | **-** | **0.0032** | **-** | **0.478** | **Y** | **0.916** | **SP** | **0.618** | **M** |
| **PEPC** | **TRINITY_DN107354_c0_g1** | **-** | **0.0252** | **Y** | **0.557** | **-** | **0.022** | **other** | **0.969** | **O** |
|  | **TRINITY_DN134009_c0_g1** | **-** | **0.0072** | **-** | **0.439** | **-** | **0.053** | **other** | **0.999** | **O** |
|  | **TRINITY_DN130243_c0_g1** | **-** | **0.0008** | **-** | **0.424** | **-** | **0.143** | **other** | **0.999** | **O** |
| **PEPCK** | **TRINITY_DN101912_c0_g1** | **-** | **0.013** | **-** | **0.461** | **Y** | **0.954** | **M** | **0.978** | **M** |
|  | **TRINITY_DN101889_c0_g3** | **-** | **0.0013** | **-** | **0.49** | **Y** | **0.870** | **M** | **0.683** | **M** |
| **PPDK** | **TRINITY_DN14534_c0_g1** | **-** | **0.0012** | **-** | **0.461** | **-** | **0.074** | **C** | **0.638** | **C** |
|  | **TRINITY_DN141134_c0_g1** | **-** | **0.0031** | **Y** | **0.528** | **-** | **0.103** | **M** | **0.512** | **C** |
|  | **TRINITY_DN87488_c0_g1** | **-** | **0.0038** | **-** | **0.435** | **-** | **0.071** | **M** | **0.618** | **M** |
| **AST** | **TRINITY_DN100628_c0_g1** | **-** | **0.0149** | **-** | **0.431** | **Y** | **0.311** | **other** | **0.556** | **M** |
|  | **TRINITY_DN102918_c1_g1** | **-** | **0.0002** | **-** | **0.434** | **Y** | **0.847** | **other** | **0.626** | **M** |
|  | **TRINITY_DN102005_c0_g1** | **-** | **0.0702** | **Y** | **0.559** | **Y** | **0.943** | **M** | **0.538** | **M** |
| **ALT** | **TRINITY_DN107143_c0_g1** | **-** | **0.0005** | **-** | **0.479** | **-** | **0.173** | **other** | **0.544** | **O** |
|  | **TRINITY_DN127408_c0_g1** | **-** | **0.0015** | **-** | **0.499** | **Y** | **0.981** | **M** | **0.530** | **M** |
|  | **TRINITY_DN100482_c0_g1** | **-** | **0.0022** | **-** | **0.442** | **Y** | **0.641** | **other** | **0.785** | **M** |
| **PC** | **TRINITY_DN105351_c0_g1** | **-** | **0.0035** | **-** | **0.458** | **-** | **0.215** | **other** | **0.992** | **O** |
|  | **TRINITY_DN15039_c0_g1** | **-** | **0.0005** | **Y** | **0.524** | **Y** | **0.544** | **M** | **0.887** | **M** |
| **Note: Sum prediction was made based on the following criteria.** | | |  | |  |  |  |  |  |  |
| **1) Proteins with both signal peptide and chloroplast transit peptide were predicted to be localized in chloroplast.** | | | | | | | |  |  |  |
| **2) Prediction with the highest probabilities was chosen for proteins with ambiguous localizations.** | | | | | | |  |  |  |  |
| **3) When the program-based prediction is contradictory to the experimentally-resolved localization of homologs in other eukaryotic organisms, the subcellular localization of the homologs was adopted.** | | | | | | | | | | |
|  |  |  |  |  |  |  |  |  |  |  |
