# Supplementary material for: The carbonate concentration mechanism of Pyropia yezoensis (Rhodophyta): evidence from transcriptomics and biochemical data
Source: BMC Plant Biol. 2020 Sep 15;20:424. doi: 10.1186/s12870-020-02629-4 (PMC7491142; doi:10.1186/s12870-020-02629-4)
Supplement: Supplementary file 6 — Additional file 6: Table S5. Primers used for qRT-PCR. [file 12870_2020_2629_MOESM6_ESM.docx]

Table S5 Primers used for qRT-PCR

| **Primer name** | **Sequence (5'to3')** |
| --- | --- |
| EIF4-F | CTGGACACTGGCAGCATCAAGG |
| EIF4-R | CATCATCAGGCATGGTGGCAGAG |
| ME-F | GGATGGGCTGTGGAACAAG |
| ME-R | TCTCGTTGCGGGAGTGAAG |
| CA-F | AGTATGGGCTCGTGGGCTTCC |
| CA-R | AGGGATGCGGCTGAACACAAAG |
| BCT-F | ATGGGCGTCGTTGAGTTTC |
| BCT-R | CGATAATGACCAGCGGTTG |
| MDH-F | AAGGTCACGGGCCACAACAAAG |
| MDH-R | GGCGAGGGACTGGACGATGG |
| PYC-F | CTACCGCAACGCTGGCACTG |
| PYC-R | CTCTTCGCTCACCGTGTGTTCC |
| PEPC-F | GAGGCGATGGTGGCGATTGC |
| PEPC-R | CTGCGACAGAATGGCGAGGTG |
| PPDK-F | GGCAAGAAGTTCGGCGACTCC |
| PPDK-R | CAGGTTGAGCACCGTGTCCATC |
| PEPCK-F | ATCTTGGACGCTATTCACAGTGG |
| PEPCK-R | AGGACTTAGGCACATTGAGGTTC |
